# The prevalence of COPD co-morbidities in Serbia: results of a national survey

**DOI:** 10.1038/npjpcrm.2014.8

**Published:** 2014-06-12

**Authors:** Ljudmila M Nagorni-Obradovic, Dejana S Vukovic

**Affiliations:** 1 Faculty of Medicine, University of Belgrade, Belgrade, Serbia; 2 Clinic for Pulmonology, Clinical Centre of Serbia, Belgrade, Serbia; 3 Faculty of Medicine, Institute of Social Medicine, Centre School of Public Health, University of Belgrade, Belgrade, Serbia

## Abstract

**Background:**

Research studies have found different prevalence rates for co-morbidities in patients with chronic obstructive pulmonary disease (COPD).

**Aims:**

The aim of our study was to investigate the prevalence of co-morbidities as well as functional limitations in subjects with COPD.

**Methods:**

The study was based on a nationally representative sample of the population of Serbia. Information on the health of the population was obtained from interviews and anthropometric measurements. In this study we analysed a total of 10,013 respondents aged 40 years or older. There were 653 subjects with COPD and 9,360 respondents without COPD.

**Results:**

Out of the 10,013 respondents, 5,377 were aged 40–59 years and 4,636 were 60 years or older. The prevalence of COPD was 5.0% in respondents aged 40–59 years and 8.3% in those aged 60 years or older; the total prevalence was 6.5%. The most prevalent co-morbidities among respondents with COPD were hypertension (54.5%) and dyslipidaemia (26.5%). The prevalence of all analysed co-morbidities was higher in respondents with COPD and the difference was highly statistically significant, except for stroke and malignancies, for which the difference was significant. Analysis showed that respondents with COPD had a higher prevalence of all analysed clinical factors (dizziness, obesity, anaemia and frailty) and functional impairments (mobility and hearing and visual impairment) compared with respondents without COPD. For those aged 40–59 years the difference was highest for mobility difficulty (four times higher prevalence in COPD patients) and anaemia (three times higher in COPD patients).

**Conclusion:**

Our analysis showed that the most prevalent co-morbidities in COPD were hypertension, dyslipidaemia, chronic renal disease and anxiety/depression. The finding suggests that health professionals should actively assess co-morbidities in patients with COPD.

## Introduction

During the last decade of the 20th century, the health status of the Serbian population was negatively affected by numerous factors such as long-lasting economic crisis, war in the surrounding countries and Serbia, and economic and diplomatic sanctions. However, in the last several years, Serbia has faced economic growth; in 2006, the gross domestic product per capita amounted to €3,354, with 5.7% growth. Also, Serbia’s literacy rate of 99.4% in the population aged 15–24 years is similar to that of other southeast European countries. The Gini index in 2006 was 28, which is not far from that of the neighbouring countries, with Bosnia and Herzegovina and Slovenia having slightly lower (27 and 26, respectively) and Macedonia, Bulgaria and Croatia slightly higher (29, 33 and 36, respectively) values.^1^ The total expenditure on health in 2006 was 8.1% of the gross domestic product.^2^

Compared with most former socialist countries, Serbia’s transition has started with a delay. The isolation and policy stagnation in the 1990s left it somewhat behind the other transition states in terms of socioeconomic development. In 2009 the income ratio between the most affluent and the least affluent regions was ~1:4 (€143:524).^3^

In chronic obstructive pulmonary disease (COPD), co-morbid conditions or co-morbidities may be defined as other serious diseases and chronic medical conditions that affect individuals who have COPD.^4^ Research studies have found different prevalence rates for co-morbidities in patients with COPD.^4,5^ Co-morbid illnesses are very important in COPD for multiple reasons: shared pathophysiological mechanisms for COPD and other chronic diseases and co-morbid illnesses may have a significant impact on the health status and utilisation of health services as well as hospitalisation of patients with COPD, and co-morbid illnesses, such as ischaemic heart disease or malignancies, could cause mortality earlier than respiratory causes. Aryal *et al.* ^5^ also pointed out that understanding clusters of co-morbid illnesses could be important for better understanding the diagnosis, therapy and prognosis of COPD. However, Schnell *et al.* ^6^ pointed out that studies on co-morbidities in COPD have typically focussed on select medical conditions such as heart failure and diabetes mellitus. These studies have largely failed to look comprehensively at many other high-priority conditions and functional limitations such as cognitive impairment and limited mobility. Functional limitations can have an important impact on the treatment of chronic conditions, and these conditions may also modify the effectiveness of COPD therapy, cause dangerous therapeutic interactions and make COPD therapies less feasible.^6^

The aim of our study was to investigate the prevalence of clinically relevant co-morbidities as well as functional limitations in subjects with COPD, and to explore whether the presence of co-morbidities and functional limitations differ according to age and smoking status.

## Materials and Methods

### Study population

**Figure 1:**
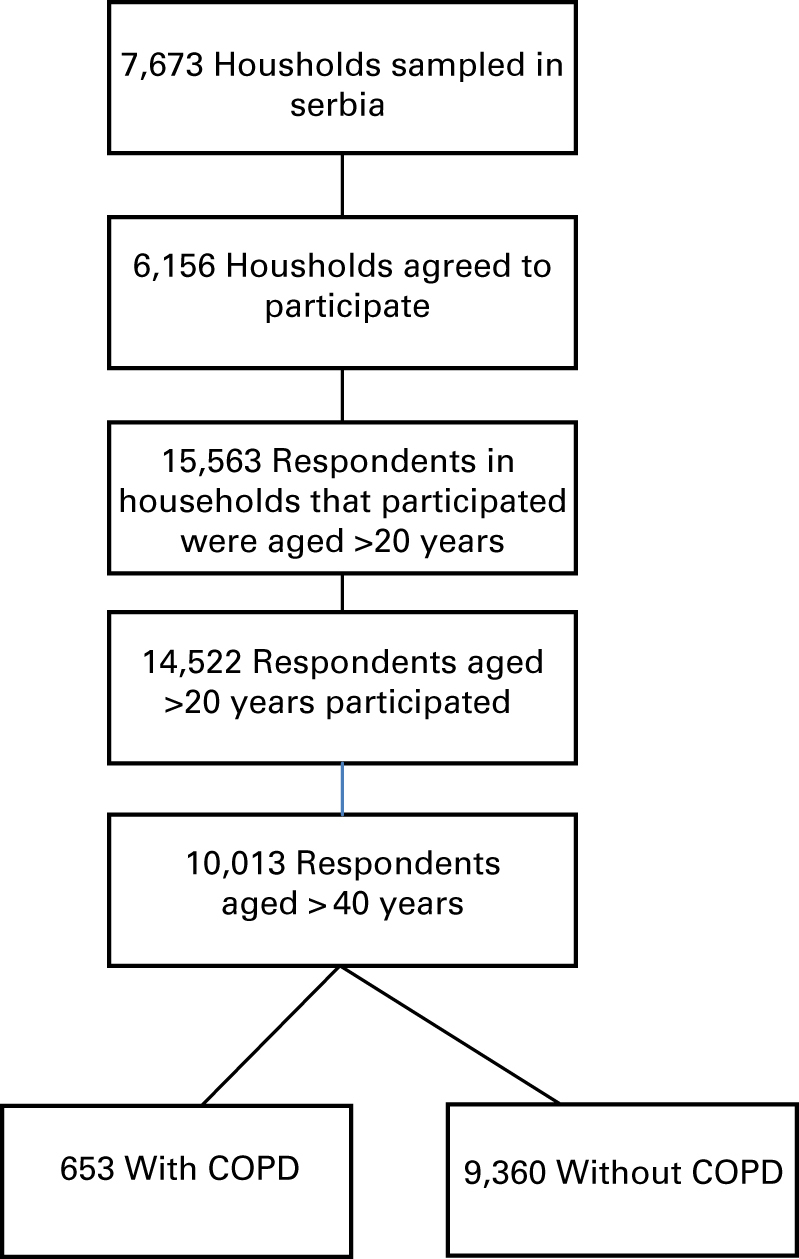
Selection of respondents.

The Institute of Public Health conducted a multipurpose health survey of the population of Serbia (excluding Kosovo) in 2006. A stratified two-stage randomised sample of all registered households in Serbia was used. The sample was selected to provide statistically reliable estimates of health at the national level and the sample was representative for the population of Serbia as a whole, as well as for urban and rural areas and for males and females. Information on the health of the population was obtained from interviews and anthropometric and blood pressure measurements. Interviews were performed in households of the respondents by a team consisting of one trained interviewer and a health worker who recorded anthropometric and blood pressure measurements. Out of 7,673 households selected, 6,156 were interviewed. The household response rate was 86.5%. In the households, there were 15,563 adults aged ⩾20 years, of whom 14,522 were interviewed, yielding a response rate of 93.3%. The overall response rate for adults was 80.5%. All adults aged 20 years and above were included, except those living in institutions (Figure 1).

All respondents were informed about the purpose of the investigation and agreed to participate. The Review Board of the Ministry of Health of Serbia and the Institute of Public Health of Serbia approved the study.

In this study we analysed a total of 10,013 respondents aged 40 years or older.

Taking into account the complexity of sampling, we included weightage in the analysis to maintain population representativeness. Weightages were calculated on the basis of estimated data for the population of Serbia in 2006.

### Variables

The classification variable to identify respondents with COPD was a self-reported history of chronic bronchitis and emphysema as measured by the following question: ‘Has a doctor ever diagnosed you to have chronic bronchitis or emphysema?’ Participants who answered positively were considered to have COPD. There were 653 subjects with COPD and 9,360 respondents without COPD, and they were analysed for the prevalence of other chronic diseases and functional limitations. The presence of other chronic diseases was measured by the following question: ‘Has a doctor ever diagnosed you to have following diseases?’ Respondents were asked to indicate all diseases diagnosed. We analysed the presence of heart attack, hypertension, stroke, asthma, malignancies, diabetes mellitus, dyslipidaemia, anxiety or depression, chronic renal disease, peptic ulcer and osteoporosis. As other clinical factors we included obesity, anaemia, frailty and dizziness.

Weight and height were measured by trained health workers following a defined protocol. Body mass index (BMI) was calculated on the basis of these values. Respondents were categorised as obese if BMI was above 30. Presence of anaemia was self-reported.

Respondents were defined as ‘frail’ if they had at least three of the following characteristics:^6^ low BMI, weakness, exhaustion and low physical activity. BMI ⩽18.5 was defined as low BMI. Weakness was defined using the following question: ‘Are you able to lift and carry 5 kilos?’ If the answer was ‘I am not able’, ‘with some difficulties’ or ‘with much difficulties’, respondents were classified as weak. Exhaustion was defined using the question ‘What is the longest distance that you can walk without stopping or feeling very tired?’ If the answer was ‘not at all’ or ‘only few steps’, respondents were defined as exhausted. Respondents were classified as having low physical activity if they estimated their physical activity as very bad or bad on a five-point scale.

Respondents were counted as having dizziness if they reported having dizziness in the previous 4 weeks.

We analysed functional limitations: hearing and visual impairment and mobility difficulty. Hearing impairment was defined as having trouble hearing when having a conversation with another person.

Visual impairment was defined as having extreme difficulty when reading text in newspapers or not able to read at all.

Mobility difficulty was defined as experiencing difficulty to walk 500 m.

The smoking status was classified as never-smokers (never smoked daily), ex-smokers (ceased smoking ⩾1 year earlier) and current daily smokers.

### Statistical analysis

The distribution of subjects with and without COPD according to socio-demographic characteristics (gender, age and education) and smoking status was analysed using descriptive statistics, the chi-square test and the *t*-test.

Differences in the prevalence of co-morbidities, clinical factors and health status factors between subjects with and without COPD were analysed using the chi-square test. Analyses were performed separately for age groups 40–59 years and above 60 years; for males and females; as well as for never-smokers, ex-smokers and current smokers.

Logistic regression analysis was performed to analyse the likelihood of occurrence of co-morbid illnesses, clinical factors and health status factors for respondents with and without COPD, adjusted for age, gender, educational level and smoking status. Tests were considered significant if *P*<0.05.

## Results

### Participants

In our study, out of 10,013 respondents 5,377 were in the age group of 40–59 years and 4,636 were aged 60 and above. The prevalence of COPD has been estimated to be 5.0% in those aged 40–59 years and 8.3% in those aged 60 and above, and the total prevalence was 6.5%. Among respondents with COPD 26.2% were current smokers and 16.8% were ex-smokers, and among those without COPD 26.6% were current smokers and 13.0% were ex-smokers, and the difference was statistically significant. The prevalence of smoking was significantly higher among those with COPD in both the age groups (Table 1).

### Prevalence of co-morbidities

Table 2 shows the prevalence of different co-morbid diseases among respondents with and without COPD.

The most prevalent co-morbidities among respondents with COPD were hypertension and dyslipidaemia.

The prevalence of all analysed co-morbidities was higher in respondents with COPD and the difference was statistically significant except for malignancies. When analysed separately in the two age groups, the prevalence of stroke and malignancies was not significantly different between respondents with and without COPD. Although the prevalence of heart attack was higher among respondents with COPD in the older age group, the difference was not significant.

When prevalence of co-morbidities was analysed separately for females and males, the prevalence of malignancies was not significantly different in respondents with and without COPD in both females and males. On the other hand, the prevalence of stroke was significantly higher only in male respondents with COPD. Peptic ulcer and heart attack were significantly more prevalent in female respondents with COPD. All other co-morbid illnesses were significantly more prevalent in both males and females with COPD. When co-morbidities were analysed separately for never-smokers, ex-smokers and current smokers, results showed that among never-smokers osteoporosis and anxiety/depression were three times more prevalent in respondents with COPD compared with those without COPD. In current smokers, osteoporosis was seven times more prevalent in respondents with COPD. Diabetes mellitus and chronic renal disease were two times more prevalent in respondents with COPD compared with those without COPD in both never-smokers and current smokers.

Among never-smokers the prevalence of stroke was not significantly different between respondents with and without COPD, but among ex-smokers stroke was more than two times more prevalent in respondents with COPD. Interestingly, in current smokers the difference was not significant (Table 3).

### Prevalence of clinical and health status factors

Analysis of clinical factors and health status factors showed that respondents with COPD had a higher prevalence of all analysed clinical factors and functional impairments compared with respondents without COPD (Table 4). For the age group 40–59 years, the difference in prevalence was highest for mobility difficulty (four times higher prevalence among respondents with COPD) and anaemia (three times higher prevalence in respondents with COPD). The prevalence of frailty was not significantly different in the age group of 40–59 years between respondents with and without COPD.

In the age group of 60 years and older, the prevalence of anaemia was more than two times higher in respondents with COPD. All other clinical and health status factors were more prevalent in respondents with COPD, except obesity.

When clinical and health status factors were analysed separately for females and males, it was seen that in females the prevalence of frailty was significantly higher among those with COPD, but among males the difference was not significant. Mobility difficulty and hearing and visual impairment were significantly higher in both females and males with COPD (Table 4).

In never-smokers, the prevalence of all analysed health status and clinical factors was higher in respondents with COPD and the difference was statistically significant. In respondents who have smoked, the prevalence of most of the analysed health status and clinical factors was significantly higher in respondents with COPD, except obesity. The prevalence of dizziness, mobility difficulty and hearing and visual impairment was almost three times higher in current smokers with COPD compared with ex-smokers, in whom it was two times higher. In current smokers with COPD, the prevalence of anaemia was five times higher than in those without COPD. In ex-smokers this difference was threefold. However, the prevalence of obesity was not significantly different between ex-smokers and current smokers with and without COPD. Among current smokers, frailty was significantly higher in subjects with COPD (Table 5).

### Logistic regression models of co-morbid illnesses and clinical and health status factors

In Table 6 results of the logistic regression analysis are presented. We analysed the odds ratios for different co-morbidities for respondents with COPD compared with those without COPD when controlling for age, gender, education and smoking status. Respondents with COPD had an almost 12 times higher likelihood of being diagnosed with asthma and 3.5 times higher likelihood of being diagnosed with anxiety/depression and osteoporosis compared with those without COPD. For osteoporosis, we additionally analysed a model that included physical activity and BMI as independent variables and we obtained an odds ratio of 3.78 (confidence interval 2.77–5.15). Results showed that the likelihood of having co-morbid illnesses was significantly higher among those with COPD for all analysed illnesses, except for heart attack, stroke and malignancies.

In Table 7 the odds ratios for clinical and health status factors for respondents with COPD compared with those without COPD when controlling for age, gender, education and smoking status are presented. Respondents with COPD had a higher likelihood of having all analysed factors compared with respondents without COPD, except frailty. The odds ratio was highest for anaemia and respondents with COPD were three times more likely to be affected by it. In the logistic regression model for obesity, physical activity was included as an independent variable.

## Discussion

### Main findings

The prevalence of COPD in our population was similar to the results obtained in other studies.^5^ Interestingly, we found a relatively high percentage of never-smokers among respondents with COPD. The high prevalence of COPD in those who have never smoked could be explained by the fact that genetic predisposition and exposure other than smoking could be important.^7^ We did not analyse passive smoking, which is also recognised as an important factor contributing to increased risk for COPD in never-smokers.^8^ Hypertension is the most prevalent co-morbidity in our respondents with COPD and is significantly higher among those with COPD compared with respondents without, even when stratified for age group and smoking status. We did not find a statistically significant difference in the prevalence of heart attack between respondents with and without COPD, even when smokers, ex-smokers and non-smokers were separately analysed. This finding could indicate that smoking is an independent risk factor for both COPD and heart attack, and that COPD itself does not increase the risk for heart attack. This was confirmed in a logistic regression analysis, which showed that the presence of COPD was not significant for the occurrence of a heart attack. The prevalence of stroke was not significantly different between respondents with and without COPD when age groups were analysed separately. On the other hand, among never-smokers the prevalence of stroke was similar in respondents with and without COPD. However, among smokers there was a significantly higher prevalence of stroke among those with COPD. After hypertension, the most prevalent co-morbidity in respondents with COPD was dyslipidaemia, which was significantly higher than among respondents without COPD even when stratified for age and smoking status. The prevalence of asthma was 10 times higher in respondents with COPD. The difference was significant even when analysis was performed separately by smoking status. Among functional limitations the most prevalent were visual and mobility impairments. Visual impairment was prevalent in respondents without COPD. The prevalence of mobility impairment was two times higher in respondents with COPD, and the difference was even higher in the younger age group.

### Strengths and limitations of this study

Our study was based on a cross-sectional design that does not allow conclusions to be drawn regarding cause–effect relations. As health survey data were used, spirometry data were not available and thus we were not able to analyse in the context of severity of COPD. The limitations of our study are related to measurement bias because diagnoses of chronic bronchitis or emphysema and lifestyle behaviours were reported by the respondent. Self-reported diagnosis of chronic bronchitis or emphysema could underestimate the prevalence of the disease because mild and moderate stages could be undiagnosed.

Also, data about co-morbidities were self-reported. However, some studies have demonstrated that self-reported data about disease could be considered valid.^9,10^

We are aware that some respondents with more severe symptoms of COPD were more likely to be diagnosed with COPD. On the other hand, those with mild symptoms could be undiagnosed by a physician and so would not be included in our study. We recognise that there is a proportion of respondents who were diagnosed with COPD but would probably not meet the spirometry criteria for COPD, and that some respondents who were not diagnosed by physicians would probably meet spirometry criteria. However, performing spirometry on such a large population sample was not feasible, and we believe that results on a representative population sample using self-reported data could be relevant.

The prevalence of self-reported diseases depends on the true prevalence of the diseases as well as the ability and knowledge of health-care providers diagnosing the disease. The threshold for seeking medical care also varies between population groups (according to gender, age, ethnicity and education level). Individuals with symptomatic chronic disease would visit health-care providers more often compared with individuals with minor or no symptomatic diseases. This would probably increase the differences in the prevalence of co-morbidities.

For frailty, we have used the modification of Fried’s original definition of frailty by including only three out of four criteria. That could possibly explain the rather small percentage of respondents classified as frail.

### Interpretation of findings in relation to previously published work

The prevalence of hypertension in respondents with COPD is similar to the results obtained by Schnell *et al.*;^6^ however, the prevalence was two times higher than that reported by Van Manenen *et al.* ^9^ The explanation for such a high prevalence is that hypertension is highly prevalent in the general population in Serbia, and 40.6% of respondents without COPD reported having hypertension.

Taking into account other studies with different results—some showing that COPD is an independent risk factor for ischaemic heart disease^11^ and others finding a lower risk for ischaemic heart disease among subjects with COPD^12^—further investigation is necessary. As our study was based on self-reported COPD, we did not have data regarding the stage of disease. Respondents with more severe stage of disease could have hypoxaemia due to chronic obstructive disease, which could influence coronary circulation and lead to a heart attack. Systemic inflammation is an extrapulmonary manifestation of COPD. A possible connection between COPD and the appearance of cardiovascular co-morbidities can be the presence of specific inflammatory cells in the formation of atherosclerotic plaques, plaque rupture and atherothrombosis.^13^

Smoking habits can be a risk for lung and systemic inflammation, oxidative stress and changes in vasomotor circulation.^12^

The PLATINO study found a significantly higher prevalence of asthma in respondents with COPD.^14^ Our results showed that the likelihood of being diagnosed with asthma was 12 times higher for respondents with COPD even when adjusting for age, gender, smoking status and education level. COPD is often misdiagnosed as asthma, leading to inappropriate treatment and suboptimal patient outcomes.^15^

Romme *et al.* found that the prevalence of osteoporosis is high in patients with COPD and we obtained similar results. Risk factors contributing to osteoporosis in COPD could be long corticosteroid therapy, overweight and old age of subjects.^16^

The prevalence of depression and anxiety in COPD was in the range of 7–80% in different studies.^17–19^ In our study, subjects with COPD were three times more likely to have anxiety or depression compared with those without COPD. Theorems relating to depression in COPD have focussed on the mechanisms associated with the complicated role of smoking, hypoxia, systemic inflammation and the impact of illness on patients’ lives.

Besides co-morbidities, some functional limitations such as cognitive impairment and limited mobility could be present in patients with COPD and affect patients’ adhering to treatment.^6^

### Implications for further research, policy and practice

In respondents with COPD, we found a significant prevalence of co-morbidities that can significantly influence the treatment and prognosis of COPD. However, it has been found that co-morbidities could be undetected and therefore not taken into account during the treatment of COPD.^20^ The finding suggests that health-care workers should actively assess co-morbidities in patients with COPD. Guidelines for management of COPD should focus on the most prevalent co-morbidities in order to enhance effective management of COPD. Development of a simple test that could be used to detect the possible presence of co-morbidities would be important for the management of COPD. An example of a simple test is the COPD Assessment Test questionnaire, which can also be used as a useful tool for assessing patients’ experience of COPD. COPD Assessment Test proved to be a practical tool for assisting primary care physicians in the identification of patients at increased risk for exacerbations.^21^

### Conclusion

Our analysis showed that the most prevalent co-morbidities in respondents with COPD were hypertension, dyslipidaemia, chronic renal disease and anxiety/depression. For management of COPD it is very important to take into account co-morbidities and functional limitations.

## Figures and Tables

**Table 1:** Socio-demographic characteristics of respondents

*Parameters*	*Total subjects*			*Age*					
				*40–59 years*			*60+ years*		
	*Non-COPD*	*COPD*	P	*Non-COPD*	*COPD*	P	*Non-COPD*	*COPD*	P
Gender (male)	4,901 (54.4%)	285 (46.6%)	<0.01	2,562 (51.4%)	110 (42.5%)	<0.01	2,339 (58.1%)	175 (49.7%)	<0.01
Mean age (s.d.), years	59.3 (12.2)	62.8 (12.4)	<0.01	49.8 (5.7)	50.4 (5.7)	0.69	71.0 (6.9)	72.0 (6.6)	<0.01
Education			<0.01			0.01			0.97
Elementary	4,584 (49.0%)	371 (56.8%)		1,655 (32.4%)	105 (39.0%)		2,929 (68.9%)	266 (69.3%)	
Secondary	3,588 (38.3%)	220 (33.7%)		2,658 (52.0%)	138 (51.3%)		930 (21.9%)	82 (21.4%)	
University	1,188 (12.7%)	62 (9.5%)		795 (15.6%)	26 (9.7%)		393 (9.2%)	36 (9.4%)	
Smoking			<0.01			<0.01			<0.01
Never-smoker	3,797 (40.6%)	206 (31.5%)		1,992 (39.0%)	78 (29.0%)		1,805 (42.4%)	128 (33.3%)	
Ex-smokers	1,222 (13.0%)	110 (16.8%)		649 (12.7%)	47 (17.5%)		573 (13.5%)	63 (16.4%)	
Current smokers	2,486 (26.6%)	171 (26.2%)		1,989 (38.9%)	117 (43.5%)		497 (11.7%)	54 (14.1%)	
Unknown	1,855 (19.8%)	166 (25.4%)		478 (9.4%)	27 (10.0%)		1,377 (32.4%)	139 (36.2%)	
Total	9,360 (100%)	653 (100%)		5,108 (100%)	269 (100%)		4,252 (100%)	384 (100%)	

Abbreviation: COPD, chronic obstructive pulmonary disease.

**Table 2:** Prevalence of co-morbid illnesses in respondents with and without COPD

*Diseases*	*Total subjects*			*Age*						*Gender*					
				*40–59 years*			*60+ years*			*Females*			*Males*		
	*Non-COPD,* N *(%)*	*COPD,* N *(%)*	P	*Non-COPD,* N *(%)*	*COPD,* N *(%)*	P	*Non-COPD,* N *(%)*	*COPD,* N *(%)*	P	*Non-COPD,* N *(%)*	*COPD,* N *(%)*	P	*Non-COPD,* N *(%)*	*COPD,* N *(%)*	P
Heart attack	508 (5.6)	50 (8.2)	<0.01	148 (3.0)	10 (3.9)	0.40	360 (8.9)	40 (11.4)	0.13	165 (4.0)	24 (7.3)	<0.01	343 (7.0)	27 (9.5)	0.11
Hypertension	3,534 (39.2)	332 (54.5)	<0.01	1,447 (29.0)	116 (45.0)	<0.01	2,087 (51.8)	216 (61.5)	<0.01	1,845 (44.9)	183 (56.1)	<0.01	1,689 (34.5)	150 (52.6)	<0.01
Stroke	339 (3.8)	33 (5.4)	0.04	82 (1.6)	6 (2.3)	0.41	257 (6.4)	27 (7.7)	0.33	136 (3.3)	13 (4.0)	0.52	203 (4.1)	21 (7.4)	<0.01
Asthma	246 (2.7)	163 (26.7)	<0.01	82 (1.6)	52 (20.1)	<0.01	164 (4.1)	111 (31.5)	<0.01	111 (2.7)	76 (23.3)	<0.01	134 (2.7)	88 (30.9)	<0.01
Malignancy	211 (2.3)	21 (3.4)	0.09	96 (1.9)	7 (2.7)	0.37	115 (2.9)	14 (4.0)	0.23	133 (3.2)	15 (4.6)	0.19	79 (1.6)	7 (2.5)	0.28
Diabetes mellitus	810 (9.0)	100 (16.4)	<0.01	258 (5.2)	36 (13.9)	<0.01	552 (13.7)	64 (18.2)	0.02	380 (9.2)	51 (15.6)	<0.01	430 (8.8)	49 (17.2)	<0.01
Dyslipidaemia	1,347 (15.0)	162 (26.5)	<0.01	685 (13.7)	60 (23.2)	<0.01	662 (16.4)	102 (29.0)	<0.01	727 (17.7)	92 (28.2)	<0.01	620 (12.7)	70 (24.6)	<0.01
Anxiety/depression	451 (5.0)	99 (16.2)	<0.01	238 (4.8)	42 (16.3)	<0.01	213 (5.3)	57 (16.2)	<0.01	276 (6.7)	63 (19.3)	<0.01	175 (3.6)	36 (12.6)	<0.01
Chronic renal disease	838 (9.3)	126 (20.6)	<0.01	375 (7.5)	42 (16.2)	<0.01	463 (11.5)	84 (23.8)	<0.01	439 (10.7)	75 (23.0)	<0.01	398 (8.1)	51 (17.9)	<0.01
Peptic ulcer	717 (8.0)	84 (13.7)	<0.01	353 (7.1)	38 (14.7)	<0.01	364 (9.0)	46 (13.1)	0.01	260 (6.3)	52 (15.9)	<0.01	456 (9.3)	32 (11.3)	0.27
Osteoporosis	334 (3.7)	79 (13.0)	<0.01	123 (2.5)	28 (10.9)	<0.01	211 (5.2)	51 (14.5)	<0.01	255 (6.2)	55 (16.9)	<0.01	80 (1.6)	24 (8.5)	<0.01

Abbreviation: COPD, chronic obstructive pulmonary disease.

**Table 3:** Prevalence of co-morbid illness in respondents with and without COPD according to smoking status

*Diseases*	*Smoking status*								
	*Never-smokers*			*Ex-smokers*			*Current smokers*		
	*Non-COPD,* N *(%)*	*COPD,* N *(%)*	P	*Non-COPD,* N *(%)*	*COPD,* N *(%)*	P	*Non-COPD,* N *(%)*	*COPD,* N *(%)*	P
Total *N*	3,797	206		1,222	110		2,486	171	
Heart attack	190 (5.0)	11 (5.3)	0.83	101 (8.3)	11 (10.1)	0.51	111 (4.5)	10 (5.8)	0.40
Hypertension	1,613 (42.5)	115 (55.8)	<0.01	498 (40.8)	65 (58.6)	<0.01	689 (27.7)	68 (39.5)	<0.01
Stroke	118 (3.1)	9 (4.4)	0.32	36 (2.9)	8 (7.4)	0.01	48 (1.9)	6 (3.5)	0.16
Asthma	97 (2.6)	62 (30.1)	<0.01	39 (3.2)	26 (23.6)	<0.01	50 (2.0)	37 (21.6)	<0.01
Malignancy	89 (2.3)	9 (4.4)	0.07	33 (2.7)	5 (4.5)	0.27	46 (1.9)	4 (2.3)	0.65
Diabetes mellitus	359 (9.5)	41 (19.9)	<0.01	125 (10.2)	14 (12.7)	0.41	139 (5.6)	19 (11.1)	<0.01
Dyslipidaemia	613 (16.2)	55 (26.7)	<0.01	209 (17.1)	33 (30.0)	<0.01	312 (12.6)	38 (22.1)	<0.01
Anxiety/depression	179 (4.7)	30 (14.6)	<0.01	55 (4.5)	17 (15.6)	<0.01	121 (4.9)	32 (18.6)	<0.01
Chronic renal disease	383 (10.1)	41 (19.9)	<0.01	119 (9.7)	20 (18.2)	<0.01	194 (7.8)	27 (15.8)	<0.01
Peptic ulcer	220 (5.8)	18 (8.8)	0.08	121 (9.9)	13 (11.8)	0.52	235 (9.5)	37 (21.5)	<0.01
Osteoporosis	154 (4.1)	32 (15.5)	<0.01	32 (2.6)	6 (5.5)	0.08	60 (2.4)	26 (15.2)	<0.01

Abbreviation: COPD, chronic obstructive pulmonary disease.

**Table 4:** Clinical factors and health status factors of respondents

*Clinical factors and health status factors*	*Total subjects*			*Age*						*Gender*					
				*40–59 years*			*60+ years*			*Females*			*Males*		
	*Non-COPD,* N *(%)*	*COPD,* N *(%)*	P	*Non-COPD,* N *(%)*	*COPD,* N *(%)*	P	*Non-COPD,* N *(%)*	*COPD,* N *(%)*	P	*Non-COPD,* N *(%)*	*COPD,* N *(%)*	P	*Non-COPD N (%)*	*COPD N (%)*	P
*Clinical factors*															
Dizziness	1,236 (13.8)	171 (28.1)	<0.01	524 (10.5)	71 (27.6)	<0.01	712 (17.1)	100 (28.5)	<0.01	755 (18.4)	111 (34.3)	<0.01	482 (9.9)	60 (21.1)	<0.01
Obesity	1,964 (23.0)	158 (27.6)	0.01	1,120 (23.0)	79 (31.1)	<0.01	844 (22.9)	79 (24.8)	0.42	1,035 (26.6)	95 (31.3)	0.08	929 (19.9)	63 (23.5)	0.15
Anaemia	316 (3.5)	61 (10.0)	<0.01	189 (3.8)	34 (13.2)	<0.01	127 (3.2)	27 (7.6)	<0.01	252 (6.1)	55 (16.8)	<0.01	63 (1.3)	6 (2.1)	0.24
Frailty	108 (1.3)	17 (3.0)	<0.01	14 (0.3)	2 (0.8)	0.16	94 (2.6)	15 (4.7)	0.02	70 (1.8)	12 (3.9)	<0.01	38 (0.8)	5 (1.9)	0.07
															
*Health status factors*															
Mobility difficulty	1,616 (18.2)	224 (37.)	<0.01	274 (5.5)	56 (21.6)	<0.01	1,342 (34.2)	168 (48.4)	<0.01	900 (22.2)	124 (38.4)	<0.01	716 (14.8)	100 (35.2)	<0.01
Hearing impairment	842 (9.4)	100 (16.3)	<0.01	119 (2.4)	12 (4.6)	0.02	723 (18.0)	88 (24.9)	<0.01	352 (8.6)	44 (13.5)	<0.01	490 (10.0)	56 (19.6)	<0.01
Visual impairment	1,283 (14.3)	152 (24.8)	<0.01	311 (6.2)	28 (10.8)	<0.01	972 (24.2)	124 (35.1)	<0.01	660 (16.1)	85 (26.1)	<0.01	623 (12.7)	66 (23.2)	<0.01

Abbreviation: COPD, chronic obstructive pulmonary disease.

**Table 5:** Clinical factors and health status factors of respondents according to smoking status

*Clinical factors and health status factors*	*Smoking status*								
	*Never-smokers*			*Ex-smokers*			*Current smokers*		
	*Non-COPD,* N *(%)*	*COPD,* N *(%)*	P	*Non-COPD,* N *(%)*	*COPD,* N *(%)*	P	*Non-COPD,* N *(%)*	*COPD,* N *(%)*	P
*Clinical factors*									
Dizziness	502 (13.2)	51 (24.8)	<0.01	135 (11.1)	22 (19.8)	<0.01	289 (11.7)	47 (28.0)	<0.01
Obesity	934 (25.3)	68 (34.2)	<0.01	321 (26.8)	31 (29.5)	0.55	445 (18.2)	39 (22.8)	0.13
Anaemia	153 (4.0)	17 (8.3)	<0.01	40 (3.3)	9 (8.2)	<0.01	71 (2.9)	26 (15.2)	<0.01
Frailty	38 (1.0)	6 (3.0)	0.01	6 (0.5)	1 (0.9)	0.55	12 (0.5)	3 (1.8)	0.03
									
*Health status factors*									
Mobility difficulty	595 (15.8)	68 (33.0)	<0.01	173 (14.2)	36 (32.7)	<0.01	196 (7.9)	44 (25.7)	<0.01
Hearing impairment	293 (7.7)	30 (14.6)	<0.01	82 (6.7)	15 (13.6)	<0.01	113 (4.6)	16 (9.4)	<0.01
Visual impairment	439 (11.6)	45 (21.8)	<0.01	139 (11.4)	22 (19.8)	<0.01	180 (7.3)	28 (16.3)	<0.01

Abbreviation: COPD, chronic obstructive pulmonary disease.

**Table 6:** Logistic regression models of co-morbid illness adjusted for age, gender, education level and smoking status

*Disease*	*Age OR (95% CI)*	*Gender (ref. male) OR (95% CI)*	*Education (ref. low) OR (95% CI)*	*Ex-smokers (ref. never-smoker) OR (95% CI)*	*Current smokers (ref. never-smoker) OR (95% CI)*	*COPD OR (95% CI)*
Heart attack	1.06 (1.05–1.06)**	0.69 (0.56–0.86)**	0.90 (0.78–1.04)	1.64 (1.27–2.11)**	1.31 (1.02–1.68)*	1.04 (0.72–1.51)
Hypertension	1.05 (1.04–1.05)**	1.61 (1.46–1.78)**	0.94 (0.88–1.01)	1.15 (1.00–1.32)*	0.78 (0.69–0.87)**	1.45 (1.19–1.75)**
Stroke	1.06 (1.04–1.07)**	0.89 (0.66–1.19)	0.84 (0.68–1.02)	1.07 (0.74–1.55)	0.98 (0.70–1.39)	1.44 (0.92–2.26)
Asthma	1.03 (1.02–1.04)**	0.93 (0.72–1.20)	0.68 (0.56–0.82)**	1.17 (0.84–1.64)	1.04 (0.77–1.40)	12.20 (9.44–15.75)**
Malignancy	1.02 (1.00–1.03)*	2.76 (2.00–3.83)**	1.14 (0.92–1.41)	1.62 (1.08–2.42)*	1.00 (0.69–1.45)	1.38 (0.83–2.28)
Diabetes mellitus	1.04 (1.03–1.05)**	1.00 (0.85–1.19)	0.88 (0.79–0.99)*	1.09 (0.88–1.35)	0.78 (0.64–0.96)*	1.72 (1.32–2.24)**
Dyslipidaemia	1.02 (1.01–1.02)**	1.59 (1.41–1.82)**	1.20 (1.10–1.31)**	1.22 (1.02–1.44)*	0.88 (0.75–1.02)	1.80 (1.45–2.23)**
Anxiety/depression	1.01 (1.00–1.02)	1.96 (1.58–2.42)**	0.73 (0.62–0.85)**	1.39 (1.04–1.87)*	1.43 (1.13–1.80)**	3.43 (2.62–4.48)**
Chronic renal disease	1.02 (1.01–1.03)**	1.39 (1.19–1.63)**	0.88 (0.79–0.99)*	1.12 (0.90–1.39)	0.97 (0.81–1.17)	1.93 (1.51–2.46)**
Peptic ulcer	1.02 (1.01–1.03)**	0.90 (0.75–1.07)	1.03 (0.92–1.16)	1.67 (1.32–2.10)**	2.05 (1.68–2.49)**	1.72 (1.31–2.26)**
Osteoporosis	1.04 (1.03–1.05)**	4.47 (3.38–5.93)**	1.15 (0.96–1.36)	0.92 (0.63–1.35)	1.12 (0.84–1.49)	3.68 (2.72–4.98)**

Abbreviations: CI, confidence interval; COPD, chronic obstructive pulmonary disease; OR, odds ratio; ref., reference.

*P<0.05; **P<0.01.

**Table 7:** Logistic regression models for clinical factors and health status factors adjusted for age, gender, education level and smoking status

	*Age OR (95% CI)*	*Gender (ref. male) OR (95% CI)*	*Education (ref. low) OR (95% CI)*	*Ex-smokers (ref. never-smokers) OR (95% CI)*	*Current smokers (ref. never-smokers) OR (95% CI)*	*COPD OR (95% CI)*
*Clinical factors*						
Dizziness	1.02 (1.01–1.03)**	2.33 (2.02–2.69)**	0.71 (0.64–0.79)**	1.24 (1.01–1.52)*	1.35 (1.15–1.59)**	2.01 (1.61–2.51)**
Obesity^a^	0.99 (0.99–0.99)*	1.28 (1.15–1.44)**	0.78 (0.72–0.85)**	1.24 (1.06–1.43)**	0.68 (0.60–0.78)**	1.28 (1.04–1.58)*
Anaemia	1.00 (0.99–1.01)	6.56 (4.83–8.91)**	1.24 (1.05–1.47)*	1.39 (0.98–1.97)	1.04 (0.79–1.38)	2.99 (2.16–4.13)**
Frailty	1.12 (1.09–1.14)**	3.36 (1.92–5.88)**	0.75 (0.50–1.13)	0.96 (0.42–2.21)	1.68 (0.88–3.20)	1.73 (0.86–3.50)
						
*Health status factors*						
Mobility difficulty	1.10 (1.09–1.10)**	2.01 (1.72–2.35)**	0.54 (0.48–0.60)**	1.48 (1.21–1.81)**	1.28 (1.07–1.54)**	2.35 (1.87–2.96)**
Hearing impairment	1.10 (1.09–1.11)**	0.80 (0.66–0.98)*	0.64 (0.56–0.74)**	0.96 (0.74–1.25)	1.26 (0.99–1.60)	1.62 (1.20–2.18)**
Visual impairment	1.06 (1.05–1.07)**	1.40 (1.19–1.64)**	0.71 (0.63–0.79)**	1.26 (1.03–1.56)*	1.13 (0.94–1.37)	1.65 (1.29–2.11)**

Abbreviations: CI, confidence interval; COPD, chronic obstructive pulmonary disease; OR, odds ratio; ref., reference.

*P<0.05; **P<0.01.

a Adjusted additionally for physical activity.
